# Peroxisome Protein Transportation Affects Metabolism of Branched-Chain Fatty Acids That Critically Impact Growth and Development of *C. elegans*

**DOI:** 10.1371/journal.pone.0076270

**Published:** 2013-09-27

**Authors:** Rencheng Wang, Marina Kniazeva, Min Han

**Affiliations:** Howard Hughes Medical Institute and Department of Molecular, Cellular and Developmental Biology, University of Colorado at Boulder, Boulder, Colorado, United States of America; The University of New South Wales, Australia

## Abstract

The impact of specific lipid molecules, including fatty acid variants, on cellular and developmental regulation is an important research subject that remains under studied. Monomethyl branched-chain fatty acids (mmBCFAs) are commonly present in multiple organisms including mammals, however our understanding of mmBCFA functions is very limited. *C. elegans* has been the premier model system to study the functions of mmBCFAs and their derived lipids, as mmBCFAs have been shown to play essential roles in post-embryonic development in this organism. To understand more about the metabolism of mmBCFAs in *C. elegans*, we performed a genetic screen for suppressors of the L1 developmental arrest phenotype caused by mmBCFA depletion. Extensive characterization of one suppressor mutation identified *prx-5*, which encodes an ortholog of the human receptor for the type-1 peroxisomal targeting signal protein. Our study showed that inactivating *prx-5* function compromised the peroxisome protein import, resulting in an increased level of branched-chain fatty acid C17ISO in animals lacking normal mmBCFA synthesis, thereby restoring wild-type growth and development. This work reveals a novel connection between peroxisomal functions and mmBCFA metabolism.

## Introduction

Lipids are known for their basic functions in energy storage, structural support of cell membranes, and adaptation to different environmental conditions [1,2]. Extensive research has indicated that they also play critical signaling and regulatory roles in various cellular and developmental events [3-8]. Like proteins and noncoding RNAs, lipid molecules are extremely diverse in their structures, but the functional specificities associated with these structural variations are poorly understood. Fatty acids (FAs), the basic components of lipids, are highly variable in their structure with varying carbon chain length, straight- or branched-chain, and saturated or unsaturated characteristics [9,10]. In particular, very little is known about the roles of monomethyl branched-chain fatty acids (mmBCFAs) that are present in animals including mammals [11-13].

**Figure 1:**
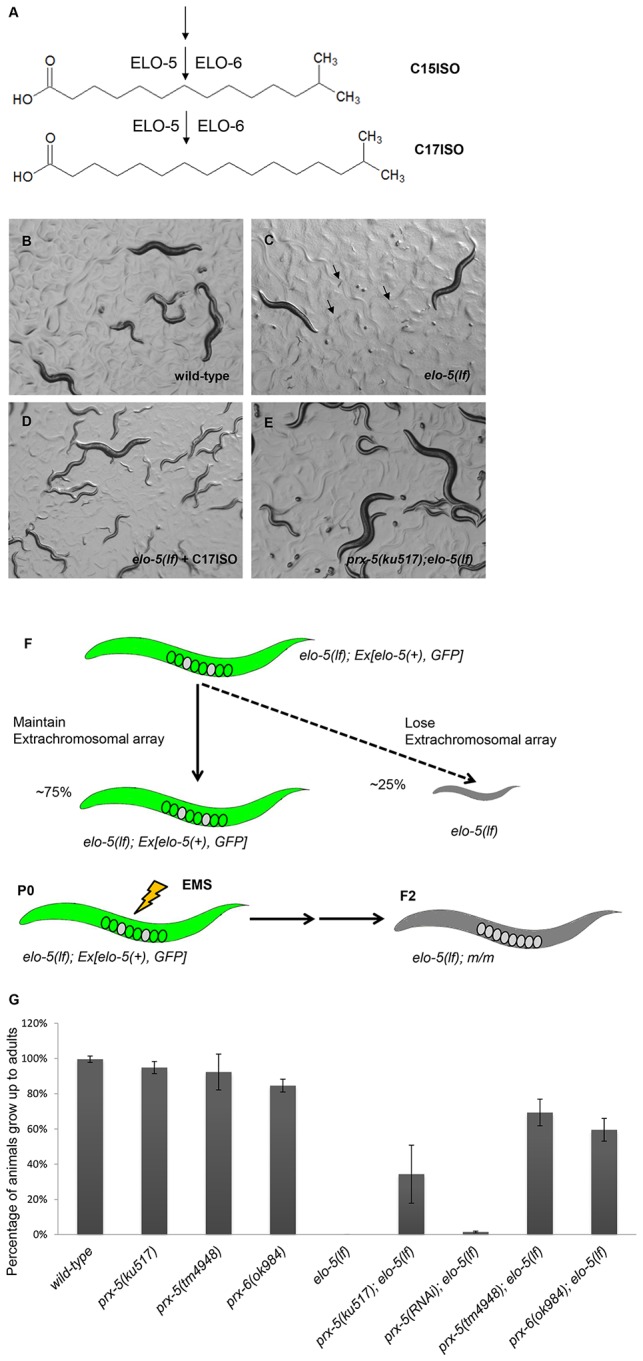
The growth defect of *elo-5*(*lf*) is suppressed by mutations in *prx-5* and *prx-6*. (A) Structure of mmBCFA C17ISO (15-methyl hexadecanoic acid) and C15ISO (13-methyl tetradecanoic acid). ELO-5 and ELO-6 are responsible for synthesis of C15ISO and C17ISO. (B-E) Microscopic images of *C. elegans* of indicated genotypes and treatments. Unlike WT (B) *elo-5*(*lf*) mutants depleted for mmBCFA after hatching display a robust L1 growth arrest phenotype (indicated by arrows) (C) that can be overcome by dietary supplementation of C17ISO (D). The growth arrest phenotype of *elo-5*(*lf*) is suppressed by the *ku517* allele (E). (F) Schematic representation of the suppressor screen. The extrachromosomal array is composed of two transgenic markers: GFP and *rol-6*(*dn*), but only GFP is shown in the cartoon for simplicity. (G) Bar graph showing percentage of animals that reached adulthood for strains with indicated genotypes. Three loss-of-function (*lf*) mutations in the *prx-5* and *prx-6* genes significantly suppressed the L1 arrest phenotype of *elo-5*(*lf*).

mmBCFAs C15ISO and C17ISO are saturated tetradecanoic and hexadecanoic fatty acids, respectively, that have a single methyl group added on the carbon next to the terminal carbon (Figure 1A). ELO-5 is a FA elongation enzyme, or elongase, specifically required for biosynthesis of mmBCFAs in *C. elegans*; disrupting *elo-5* function by a deletion mutation or by RNAi treatment causes dramatic and specific reduction of the levels of C15ISO and C17ISO in *C. elegans* (in the absence of dietary mmBCFAs) [11]. Eliminating *elo-5* function causes several developmental defects including robust developmental arrest at the first larval stage (L1 arrest), all of which can be overcome by dietary supplementation of chemically synthesized C15ISO or C17ISO (Figure 1B-D) [11,14]. Further study showed that mmBCFAs are incorporated into specific phospholipids by an acyl-CoA synthetase (ACS-1), which has a profound influence on IP_3_ signaling and membrane dynamics during early embryogenesis [15]. Recent work from our lab discovered that an mmBCFA-containing sphingolipid, d17iso-glucosylceramide, mediates the role of mmBCFAs in promoting postembryonic growth and development by activating the TORC1 signaling pathway in the intestine [16].

The peroxisome is an important organelle in all eukaryotic cells. It contains enzymes that are essential for multiple metabolic pathways, including amino acid metabolism, hydrogen peroxide regulation and lipid metabolism. Peroxisomal proteins are synthesized in the cytosol and require an import mechanism to execute their metabolic functions in the peroxisome. The peroxisomal import mechanism involves multiple proteins, including the peroxisomal membrane proteins and PTS receptor. In human, receptor PEX5, which has an ortholog PRX-5 in *C. elegans*, interacts directly with proteins containing the type 1 peroxisomal targeting signal (PTS-1) [17]. Peroxisomal matrix proteins containing PTS-1 on their C-terminus are synthesized on free polyribosomes in the cytosol, and then recognized by PEX5 through its interaction with the PTS-1. PEX5 then transports proteins with PTS-1 into the peroxisome matrix [18]. The peroxisomal import mechanism is essential for peroxisomal functions as it has been shown that inactivating the peroxisomal import mechanism compromises peroxisomal metabolism and causes accumulation of metabolites in humans [19].

In this study, we identified a connection between mmBCFA metabolism and peroxisomal protein transport through a search for suppressor mutations of the L1 arrest phenotype caused by a loss-of-function (*lf*) mutation in *elo-5*. Here we describe the identification and characterization of mutations in *prx-5* that disrupt the peroxisomal import mechanism and alter mmBCFA metabolism in *C. elegans*. This change, mostly likely through reducing peroxisome-involved degradation of mmBCFAs, is able to compensate for the loss of the major C17ISO biosynthesis enzyme.

## Materials and Methods

### General *C. elegans* maintenance and strains

All *C. elegans* strains were maintained at 20°C on nematode growth media (NGM) with OP 50 bacteria according to the standard protocol. The wild-type strain used was the N2 Bristol. The following mutant strains were used in this study: *elo-5*(*gk208*)IV*, prx-5*(*ku517*)II*, prx-5*(*tm4948*)II*, prx-6*(*ok984*)V*, dpy-10*(*e128*)*; unc-4*(*e120*)II, maoc-1(ok2645)II, *dhs*-28(tm2581)X and *daf-22*(*ok693*)II. *elo-5*(*gk208*) was originally obtained from the *C. elegans* Gene Knockout Consortium. The *prx*-5(tm4948)II and *dhs*-28(tm2581)X deletion strains were obtained from the National Bioresource Project in Japan. The Hawaiian strain (CB4856) was used for SNP mapping [20]. The strain containing [P_HSP16/2_:GFP-SKL] was kindly provided by M. Driscoll. The *elo-5*(*gk208*) strain and mutants containing the *elo-5*(*gk208*) allele were maintained on OP 50 bacteria supplemented with the mmBCFA-producing *Stenotrophomonas maltophilia* bacteria.

### *elo-5*(gk208) suppressor screen

An *elo-5*(*gk208*) mutant containing an *elo5*(*+*) rescuing transgene and visible markers, [*elo-5*(*gk208*);*Ex*[*elo-5(+*)*,rol-6 (su1006*)*II, Pelo-5:GFP*], was first constructed for the screen. L4-staged worms (generation P_0_) grown in the presence of 1 mM C13ISO were mutagenized by EMS. Their first and second generation progeny, F_1_ and F_2,_ were maintained as a group until the F_2_ animals grew to the L4 stage. Among F_2_ animals, non-roller and GFP-negative worms, presumably not containing the rescuing transgene, were singled out and plated on OP 50 plates without mmBCFA supplementation. Suppressor candidates were determined by the ability of their progeny to survive on these plates. From ~9000 haploid genomes, four suppressor mutants were isolated. Mapping analyses indicated that they are mutations in at least three different genes. *ku517* was mapped to chromosomal II and outcrossed five times before further analysis.

### Gas chromatography (GC) to analyze fatty acid composition

A mixed population of worms was collected from plates with M9 buffer, rinsed 3-4 times, pelleted, freeze-dried, and weighed, in preparation for lipid extraction. Collected samples were mixed with methanol with 2M KOH for 5 min at room temperature for methylation; FA methyl esters were extracted using hexane and subjected to GC analysis as described previously [11]. The abundance of mmBCFAs was analyzed by calculating the area of their peaks on the GC profile. For each sample, three replicates were assayed. The average and standard deviation (s.d.) were calculated using Excel. Statistical significance was determined using the student’s T-test.

### Position cloning of *prx-5*

**Figure 2:**
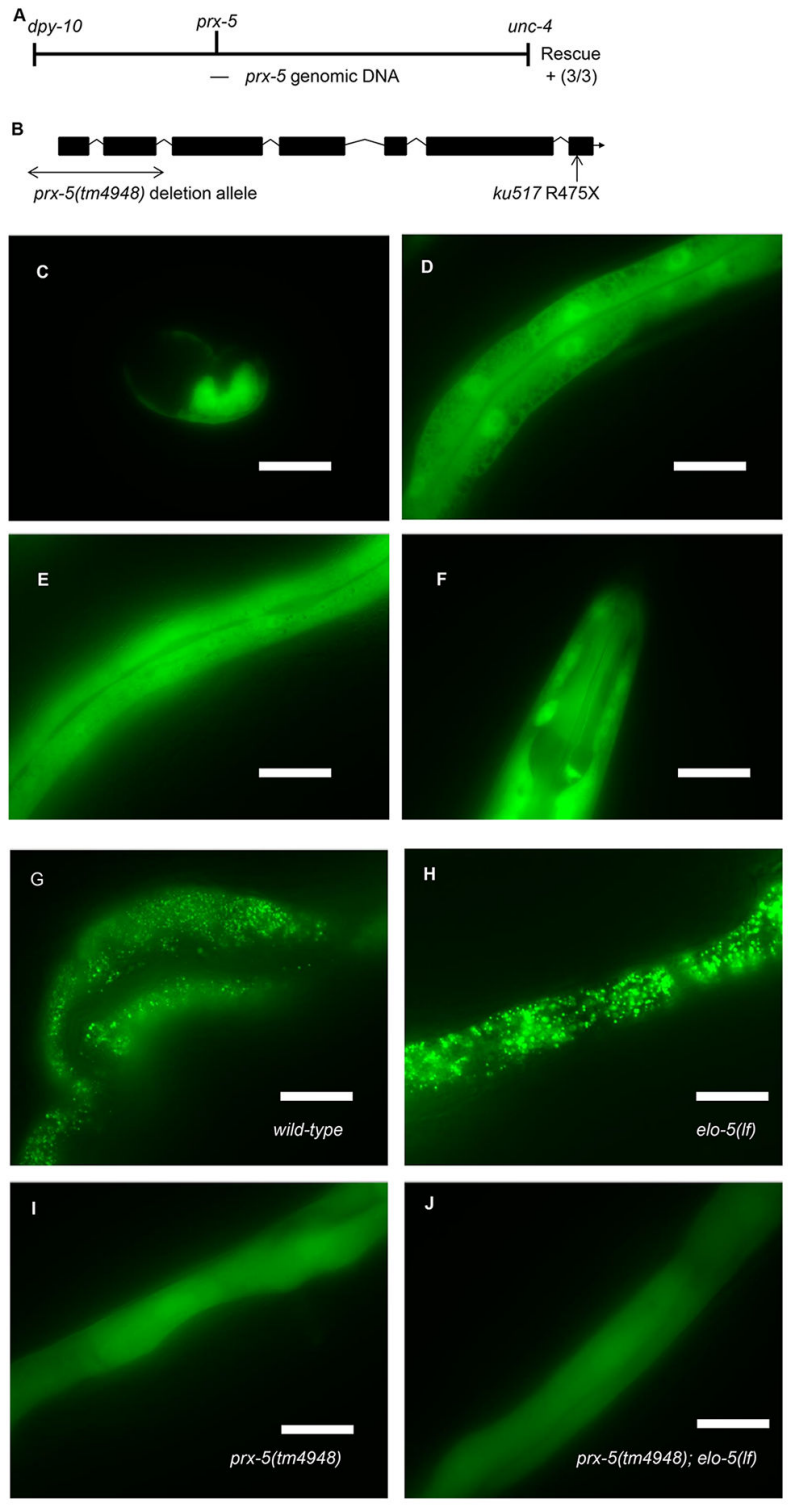
Positional cloning and analysis of *prx-5* defined by the *ku517* mutation. (A) Schematic representation of 2 centimorgan (cM) region of chromosome II containing the *ku517* mutation. The position of *ku517* relative to the *dpy-10* and *unc-4* genes was determined by three-point mapping. The location of a 7 kb genomic DNA clone able to rescue the *ku517* allele (3 out of 3 transgenic lines) is also indicated. (B) Structure of the *prx-5* gene. There are two potential cDNA isoforms differing by 6 nucleotides (wormbase.org). The C to T substitution in *prx-5*(*ku517*) results in a premature stop codon terminating the encoded protein at amino acid residue 475. The double-arrow indicates the 437 bp deletion in *prx-5*(*tm4948*) allele. (C-F) Fluorescence images showing the broad expression pattern of a *P*_*prx-5*_ *:GFP* fusion protein in *C. elegans*. The GFP reporter was observed in the embryo (C), intestine (D), hypodermis (E) and neurons (F). (G-J) Images showing the distribution of the GFP-SKL reporter that is the readout of peroxisomal import activity [17]. GFP was localized in peroxisomes as indicated by the punctate pattern in both wild type and *elo-5*(*lf*) mutants (G and H). In *prx-5*(*tm4948*), GFP is dispersed throughout the cytoplasm, indicating a defect in peroxisomal import (I and J). Bars, 25 µm.

A SNP mapping method [21] was used to locate *ku517* to the middle of chromosome II. The mutation was further mapped to a small region between *dpy-10* and *unc-4* using the three-point mapping method (Figure 2A). Recombinants were generated by crossing *ku517;elo-5*(*gk208*) to the *dpy-10*(*e128*);*unc-4(e120*);*elo-5*(*gk208*) strain. In F_2_ worms, Unc non-Dpy and Dpy non-Unc recombinants were isolated. Of these recombinants, 13/16 Unc non-Dpy and 1/8 Dpy non-Unc recombinants had the *ku517* suppressor mutation.

By applying whole genome sequencing [22] and subsequent data analysis [23], multiple DNA lesions were identified within the region. Between *dpy-10* and *unc-4*, only four homozygous DNA lesions in the coding region were identified and only a C to T lesion in the coding region of the *prx-5* gene is consistent with the three-point mapping data. In addition, only this *prx-5* mutation is present in the *ku517; elo-5*(*gk208*) double mutant but not in the *elo-5*(*gk208*) single mutant. This substitution is predicted to change a codon for *Arg 475* to a stop codon, resulting in a 26 amino acid truncation of the PRX-5 protein (Figure 2B). Genomic DNA of *prx-5* was cloned and injected along with *sur-5::GFP* (90 ng/mL) into the *ku517* mutants. The restoration of the mutant phenotype was observed in the progeny.

### RNAi analysis

RNAi by feeding and injection were performed using standard methods [24,25]. Bacterial strains were obtained from the *C. elegans* genome-wide RNAi feeding library (Geneservice). For feeding RNAi, eggs were collected from the indicated *C. elegans* strains by hypochlorite treatment and spotted on the bacterial lawn made of RNAi producing bacteria as their food source at 20°C. Controls were fed with HT115 carrying the empty pPD129.36 plasmid. For injection RNAi, T7 oligo (5’-CGTAATACGACTCACTATAG-3’) was used to amplify the DNA fragments with T7 promoter at both ends. PCR products were directly used as templates in transcription reactions to prepare dsRNA with an *in vitro* transcription kit (MEGAscript T7 from Invitrogen™). Products of *in vitro* transcription were analyzed by agarose gel electrophoresis to confirm their size and concentration.

### Assay for PRX-5 import function

Strains containing [P_HSP16/2_:GFP-SKL] were used to analyze PRX-5 function in importing peroxisomal matrix proteins, following the procedures described in [17].

### DNA constructs for transgenes

The *prx-5P::GFP* transcriptional reporter construct was generated by PCR amplification of 3 kb upstream of the *prx-5* initiation codon from N2 genomic DNA and cloned into GFP vector pPD95.77 (gift from A. Fire). Primers: F- BamHI 5’- GAGGGGCTCAAGGAGTTGGTC -3’, R- KpnI 5’- TCTGTAAAAAATTGAAGAATTCGAGAG -3’. The rescue construct of *prx-5* was generated by PCR amplification of the genomic fragment of *prx-5*, including 3.1kb upstream of initiation codon of *prx-5* and 0.4kb downstream of its stop codon. Primers: F – BamHI 5’- GAGGGGCTCAAGGAGTTGGTC -3’. R - KpnI 5’-CGTTTGGAAGAGGGTGGCG -3’. Expression construct of *elo-6* was generated by PCR amplification of a 4.5kb genomic fragment, covering 1.4kb upstream of start codon of *elo-6* and 1.1kb downstream of its stop codon. Primers: F - SalI 5’- GGCGATTGTTGATTGTTGGTTTC -3’. R-NotI 5’- GCCGAGTGTTAGGAGGAGATAGAAC -3’.

### Microscopy

Fluorescent and Nomarski optics of a Zeiss Axioplan2 microscope and Zeiss AxioCam MRm camera were used to analyze GFP expression and abnormalities in phenotypes. Phenotypes on plates were observed through a Leica MZ16F dissecting microscope and pictures were taken with a C4742-95 CCD camera.

### Examining mRNA level by real-time quantitative PCR (qPCR)

Total RNA was isolated from asynchronous, well-fed worms with TRIzol reagent (Invitrogen) following the manufacturer’s procedure. Total RNA was reverse transcribed with an oligo-dT primer, and subjected to quantitative PCR using Rotor-Gene RG-3000 (Qiagen). All qPCR data was normalized to the average Ct of a housekeeping gene, *rpl-26*.

## Results

### Isolation of suppressor mutations of the growth defect associated with *elo-5*(lf)

Previous work in our lab has shown that mmBCFAs C15ISO and C17ISO are required for normal *C. elegans* development. When the fatty acid elongase gene *elo-5* is mutated in parental animals, the resulting hatched worms uniformly arrest at the first larval (L1) stage. This robust larval growth and developmental defect can be overcome by dietary supplementation of mmBCFAs [11,14] (Figure 1A-D). To identify genes that may be involved in the metabolism of mmBCFAs or in a cellular pathway regulating mmBCFA-mediated functions in development, we performed a genetic suppressor screen, modified from our previous study [20]. We looked for mutations that would rescue the growth of animals containing a loss-of-function (*lf*) mutation as a result of deletion in *elo-5* gene, *gk208*, without mmBCFA supplementation. Hereafter, *elo-5*(*gk208*) is referred to as *elo-5*(*lf*).

For this screen, we generated a strain that was homozygous for *elo-5*(*lf*) and carried an extrachromosomal array containing copies of wild-type *elo-5* and two markers (Figure 1F; Materials and Methods). Progeny that did not inherit the extrachromosomal array and were maintained without C17ISO supplement uniformly arrested their development at L1 stage. The worms carrying the array were mutagenized by EMS and their F2 progeny that did not contain the extrachromosomal array but developed to fertile adults were singled out. These animals potentially contained suppressor mutations that permitted the *elo-5*(*lf*) animals to overcome the L1-arrest phenotype (Figure 1F). After screening ~9000 haploid genomes, we isolated 4 alleles, among which *ku517* was extensively characterized. The *ku517; elo-5*(*lf*) double mutant was able to grow indefinitely without dietary supplementation of C17ISO (Figure 1E and 1G). Thus, *ku517* may carry a mutation that alters either mmBCFA metabolism, or a regulatory pathway, that bypasses the requirement for these mmBCFAs in development.

### *ku517* is a loss-of-function mutation in *prx-5*, an ortholog of the human receptor for the type-1 peroxisomal targeting signal proteins

Using single nucleotide polymorphism (SNP) mapping [21], conventional three-point mapping methods and the whole genome sequence analysis, we determined that *ku517* is a C-T substitution that changes CGA coding for Arginine 475 to a TGA stop codon in the *prx-5* coding sequence (Figure 2B) (Materials and Methods). Therefore, the *ku517* mutant strain is expected to express the PRX-5 protein with a 26 amino acid truncation at the C-terminus, and the level of this mutant protein may be drastically reduced by the nonsense-mediated mRNA decay system [26]. We then found that a wild-type *prx-5* transgene was able to rescue the suppression phenotype of *ku517* in the *prx-5*(*ku517*)*; elo-5*(*lf*) double mutant, reverting it to larval arrest (100% L1 arrest, n>100). This confirmed that the Arg475Stop mutation in *prx-5* is the mutation responsible for the growth of *elo-5; ku517.*

We further acquired a 437-bp deletion mutation of *prx-5* [*prx-5(tm4948*)] and found that this deletion allele could also suppress the L1 arrest phenotype of *elo-5*(*lf*). Approximately 75% of *prx-5*(*tm4948*);*elo-5*(*lf*) double mutants were able to grow to fertile adults without dietary C17ISO supplement. We then used RNA interference (RNAi) to inactivate *prx-5* in *elo-5*(*lf*) mutants, but only observed a very weak suppression effect (Figure 1G). The lack of a strong suppression effect was likely due to the ineffectiveness of RNAi on this gene. The combined results indicate that loss of *prx-5* function suppresses the larval growth defect caused by disrupting the function of mmBCFA specific elongase ELO-5. We hypothesized that PRX-5 may be involved in metabolism of mmBCFA or a signaling pathway that regulates mmBCFA-mediated functions in development.

To examine the expression pattern of *prx-5*, we generated a transcriptional GFP reporter (Materials and Methods) and observed that the P*prx-5*:*GFP* is expressed at all developmental stages in many tissues including the intestine, hypodermis and neurons (Figure 2C-F).

### *prx-5*(lf) mutation compromises peroxisomal import function

To examine the impact of *prx-5*(*lf*) on peroxisomal import function, we analyzed the distribution of a reporter protein containing a GFP fused with a type-1 peroxisomal targeting signal SKL (Serine-Lysine-Leucine) at the C-terminus (GFP-SKL), which interacts directly with receptor PRX-5 [17]. This reporter was expressed in a transgenic strain carrying the P_HSP16/2_:GFP-SKL construct behind a heat-shock promoter (Figure 2G-J). After a heat-shock and recovery, GFP-SKL was found to be imported from the cytosol into peroxisomes showing clear, punctate fluorescence in multiple tissues in wild-type and *elo-5*(*lf*) animals (Figure 2G-H). In contrast, the GFP signal lost the punctate pattern and was distributed throughout the cytoplasm in *prx-5*(*tm4948*) and *prx-5*(*tm4948*)*; elo-5*(*lf*) mutants (Figure 2I-J). This observation indicated that peroxisomal proteins failed to be imported into peroxisomes and were thus retained in the cytoplasm.

This defect appears to be more severe than that from the reported RNAi analysis [17], supporting our suggestion that *prx-5*(*RNAi*) has a weak loss of function effect. The results from both studies support the conclusion that *prx-5* is required for peroxisomal import of peroxisomal matrix protein containing PTS-1 signal in *C. elegans*.

### Compromising peroxisomal import functions changes mmBCFA metabolism

Because *prx-5*(*lf*) mutations disrupt peroxisome transport that is critical for basic peroxisomal functions, we asked whether suppression of the L1 arrest phenotype of *elo-5*(*lf*) is a result of interrupting general peroxisomal function rather than an undefined specific function of *prx-5*. We thus tested the effect of inactivating *prx-6* in *elo-5*(*lf*) mutants. PRX-6 is predicted to be an ortholog of PEX6 in human, which is an AAA-ATPase responsible for recycling the peroxisomal matrix protein receptor back into the cytosol [27]. We found that a deletion allele of *prx-6, prx-6*(*ok984*), also suppressed the L1 arrest phenotype of *elo-5*(*lf*). About 60% of *prx-6*(*ok984*)*; elo-5*(*lf*) double mutants were able to grow to fertile adults without dietary supplementation of C17ISO (Figure 1G). The suppression level is comparable to that of *prx-5*(*ku517*)*; elo-5*(*lf*) and *prx-5*(*tm4948*)*; elo-5*(*lf*). This result supports the idea that disrupting the functions of peroxisome import mechanism leads to the suppression of the *elo-5*(*lf*) phenotype. The absence of genetic mutations and ineffectiveness of RNAi prevented us from examining more peroxisomal genes.

**Figure 3:**
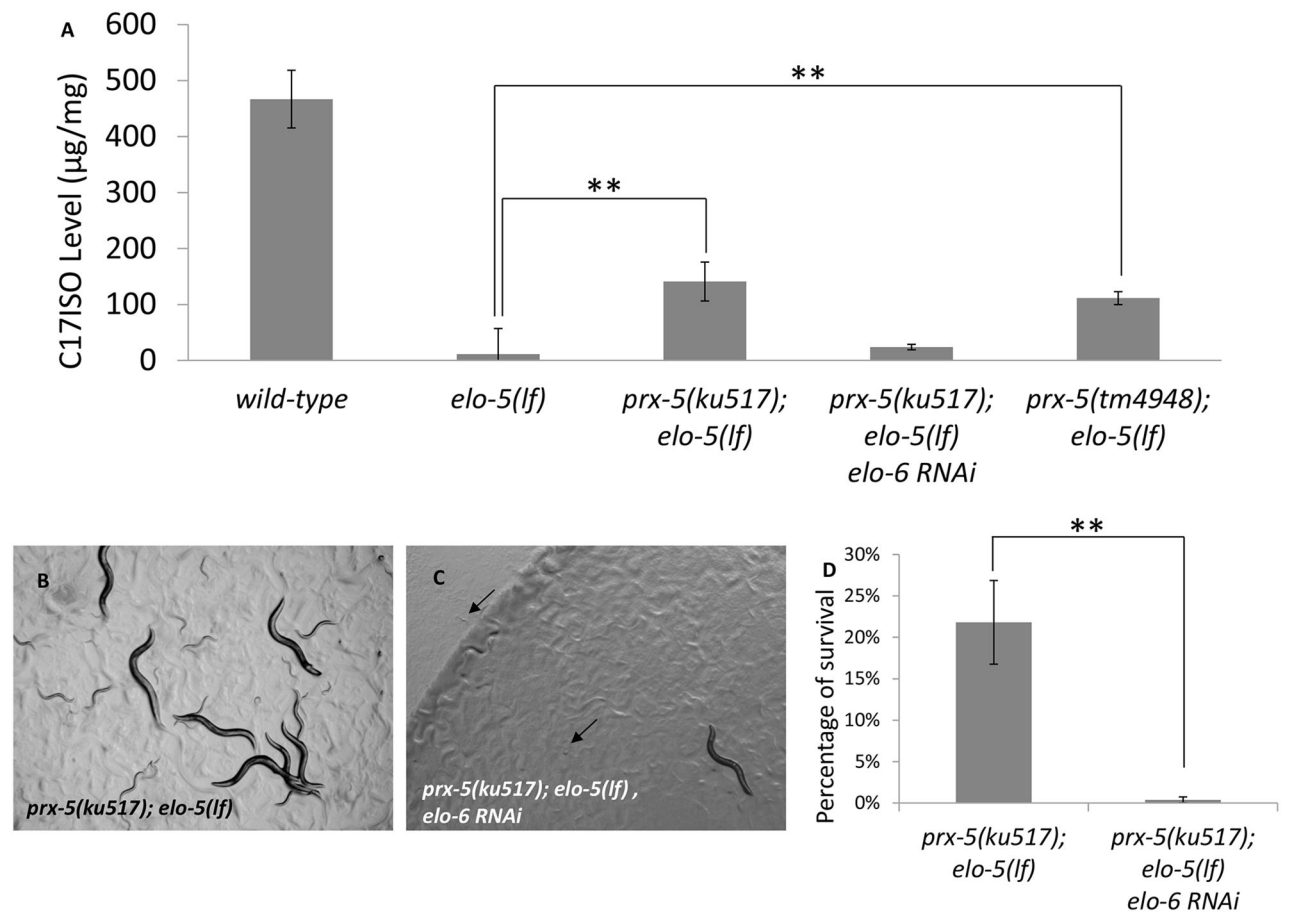
*prx-5*(*lf*) increases mmBCFA C17ISO level in *elo-5*(*lf*) mutant and its suppression depends on the *elo-6*. (A) C17ISO levels in worms of indicated genotypes cultured without mmBCFA supplementation. C17ISO concentration is dramatically decreased in *elo-5*(*lf*) animals compared to wild-type worms, and partially recovered in two suppressor mutants *prx-5*(*ku517*)*; elo-5*(*lf*) and *prx-5*(*tm4948*)*; elo-5*(*lf*). Three replicates were done for each sample. Data are presented as average concentration with corresponding standard deviation (s.d.). (B-C) Microscopic images of *C. elegans* of indicated genotypes and treatments. *prx-5*(*ku517*) overcame the L1 arrest of *elo-5*(*lf*) (B), but this suppression was reversed when *elo-6* was knocked down by RNAi (C). (D) Bar graph showing percentage of animals that reached adulthood for *prx-5*(*ku517*)*; elo-5*(*lf*) with or without *elo-6* RNAi.

To determine whether compromising peroxisomal function causes changes in mmBCFA metabolism or influences a signal pathway regulating mmBCFA-mediated functions in development, we examined the fatty acid composition of these suppressor mutants by gas chromatography (GC) (Materials and Methods). We found that both *prx-5*(*ku517*)*; elo-5*(*lf*) and *prx-5*(*tm4948*)*; elo-5*(*lf*) double mutants had a higher level of mmBCFA C17ISO when compared to that in the *elo-5*(*lf*) single mutant, albeit that the C17ISO level was still significantly below that observed in wild-type (Figure 3A). This result suggests that the suppression may be due to changes in mmBCFA metabolism.

### Suppression of *elo-5*(lf) by *prx-5*(lf) depends on another fatty acid elongase, *elo-6*

Our previous studies showed that *elo-6* is also involved in mmBCFA biosynthesis through its role in elongating C13ISO FA to C17ISO FA [11,14]. Therefore, it is possible that ELO-6 permits a low level of synthesis of C17ISO in the absence of ELO-5. Although the ELO-6 dependent synthesis of C17ISO is not sufficient to rescue the developmental defects in *elo-5*(*lf*), it could be sufficient in the *prx-5*(*lf*)*; elo-5*(*lf*) double mutants. If that is the case, and if the suppression of the L1 arrest phenotype of *elo-5*(*lf*) is mainly attributed to the increase in C17ISO level, then the suppression effect is expected to depend on intact ELO-6 activity. We thus applied *elo-6* RNAi to *prx-5*(*ku517*)*; elo-5*(*lf*) double mutants and observed that the suppressor effect of *prx-5*(*lf*) was largely eliminated. More than 99% of F1 progeny from *elo-6*(*RNAi*) treated *prx-5*(*ku517*)*; elo-5*(*lf*) double mutants were arrested at the first or second larval stages without dietary supplementation of C17ISO (Figure 3B-D). Furthermore, GC analysis showed that *elo-6*(*RNAi*) caused a decrease of the C17ISO level in *prx-5*(*ku517*)*; elo-5*(*lf*) double mutants (Figure 3A). These results indicate that disruption of peroxisomal import functions suppresses *elo-5*(*lf*) growth defect by increasing the endogenous level of mmBCFA C17ISO. This may be achieved either by enhancing ELO-6-dependent biosynthesis of C17ISO or by decreasing the degradation of the ELO-6 dependent C17ISO that may be synthesized at a low level in the *elo-5*(*lf*) mutant.

### *prx-5*(*lf*) likely decreases mmBCFA degradation

**Figure 4:**
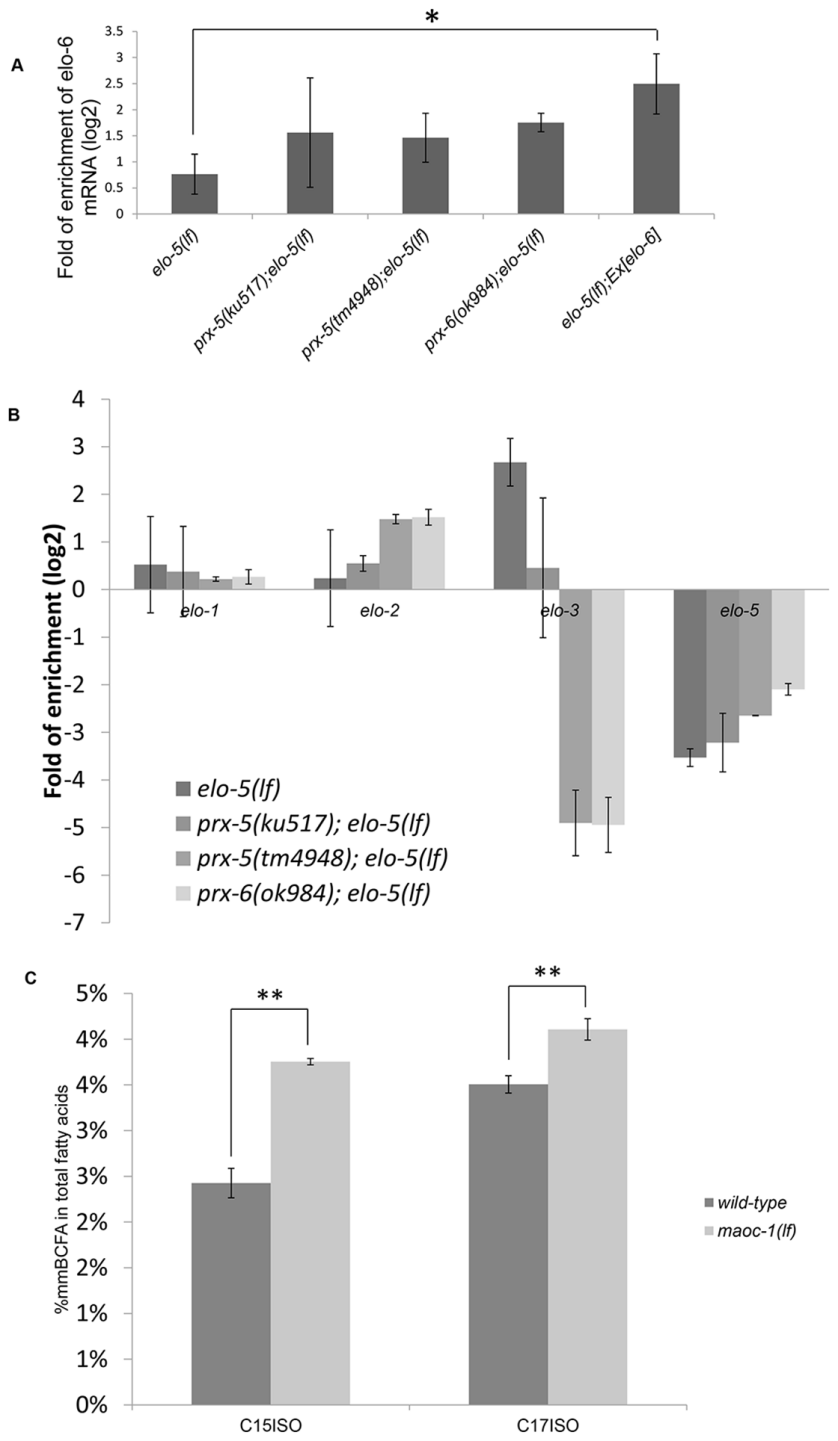
*prx-5/6* mutations likely cause a decrease in mmBCFA degradation. (A) qRT-PCR data showing mRNA levels of the *elo-6* gene in worms of indicated genotypes. *elo-5*(*lf*)*; Ex*[*elo-6*] is a transgenic strain that significantly overexpressed the *elo-6* gene and was not sufficient to suppress the L1 arrest phenotype of *elo-5*(*lf*). The data suggest that a higher level of *elo-6* expression is not likely the cause of the suppression. (B) qRT-PCR data showing mRNA levels corresponding to several other FA elongation enzymes in worms of indicated genotypes. Since the *elo-5* mRNA being measured is from the *elo-5*(*gk208, deletion*) allele, the significant decrease in *elo-5* mRNA level is likely due to mutation-induced mRNA degradation. (C) Comparison of mmBCFA composition in the indicated strains. Percentage of C15ISO and C17ISO has a statistically significant increase in the *maoc-1*(*lf*) mutant.

The increased level of C17ISO in the *prx-5*(*lf*)*; elo-5*(*lf*) double mutant, compared to the *elo-5*(*lf*) single mutant, could conceivably be caused by an increase in FA synthesis. Logically, it may be due to either a general increase in the expression of FA synthesis enzymes or a specific increase in the expression of one FA synthesis enzyme that has biochemical function similar to ELO-5. We thus examined the mRNA level of several FA elongases involved in the elongation of fatty acids with different efficiency and specificity [6,11,28-30]. Using quantitative RT-PCR (qRT-PCR), we examined the mRNA level of *elo-1, elo-2, elo-3, elo-5* and *elo-6* genes in wild-type, *elo-5*(*lf*), *prx-5*(*ku517*)*; elo-5*(*lf*), *prx-5*(*tm4948*)*; elo-5*(*lf*) and *prx-6*(*ok984*)*; elo-5*(*lf*) animals. The data presented in Figure 4B indicate that the suppression mutations in *prx-5* and *prx-6* do not cause a significant and general increase in the mRNA levels of FA elongases. Interestingly, the mRNA level of *elo-3* is significantly decreased in two of the three suppressor mutants we observed, and the two *prx-5* mutants (*ku517* and *tm4948*) behave differently. It is not clear what is the cause of the potential down-regulation of *elo-3* by these peroxisome defective mutations. The difference between the two *prx-5* mutations in this regard, possibly caused by differences in the genetic backgrounds of these two strains, does suggest that the change in *elo-3* mRNA level is not specifically linked to the suppression effects of these mutations.

We found that, when compared to wild type, the mRNA level of *elo-6* was significantly higher in the three double mutants, as well as in the *elo-5*(*lf*) single mutant (Figure 4A). This increase in *elo-5*(*lf*) is consistent with the previous finding that *elo-6* may be subjected to feedback regulation by C17ISO [14]. Although the *elo-6* mRNA level in three *prx*(*lf*)*; elo-5*(*lf*) double mutants appeared to be higher than in the *elo-5*(*lf*) single mutant, the difference was not statistically significant. In this analysis, we did not observe consistent changes in mRNA levels of other tested FA elongation enzyme genes (Figure 4B), consistent with the idea that the expression of FA elongases is not prominently affected by compromising peroxisomal import functions.

Since ELO-6 plays an essential role in synthesizing a low level of mmBCFA in *prx-5*(*lf*) in the absence of ELO-5, it is conceivable that the increase in C17ISO level in the *prx-5*(*lf*) mutant is due to increased *elo-6* activity. If that were the case, over expressing *elo-6* alone would likely to suppress *elo-5*(*lf*) L1 arrest. We thus generated transgenic animals that carried an extrachromosomal array containing multiple copies of wild type *elo-6* with its native promoter. By qPCR, we confirmed that the *elo-6* transgene was transcribed at a level significantly higher than that in the suppressor mutants (Figure 4A). Expression of this transgene failed to overcome the growth defect of *elo-5*(*lf*); 100% of *elo-5*(*lf*) animals carrying this transgene were arrested at L1 stage without dietary supplementation of C17ISO (n>400). Therefore, the suppression of *elo-5*(*lf*) by *prx-5*(*lf*) is not likely due to an increased expression of *elo-6*.

We then asked whether a decrease in mmBCFA degradation could be the key cause of the suppression effect of *prx-5*(*lf*). β-oxidation of fatty acids is one of the many conserved metabolic functions associated with the peroxisome [31]. In *C. elegans*, MAOC-1/hydratase, DHS-28/dehydrogenase and DAF-22/thiolase were reported to play important roles in the peroxisomal β-oxidation pathway [32]. If the peroxisome is responsible for the degradation of mmBCFA C17ISO, disruption of peroxisomal fatty acid β-oxidation may increase the C17ISO level and suppress the phenotype of *elo-5*(*lf*). We then applied RNAi to knock down *maoc-1, dhs-28* and *daf-22* and found the treatments were not able to suppress the developmental defect of *elo-5*(*lf*). Further analysis using single, double and triple mutants of these genes also generated negative results, indicating that mutating these genes is not sufficient to rescue the phenotype of *elo-5*(*lf*).

We thus analyzed the fatty acid profiles of these single mutants. We found that a deletion mutant of *maoc-1* displayed a significant increase in the level of C15ISO (~55%) and a small but still significant increase in the level of C17ISO (~17%) in total lipid extracts, over that in samples from wild type (Figure 4C). This increase suggests that the MAOC-1-involved β-oxidation pathway contributes to the degradation of mmBCFA, but that the increase is not sufficient to suppress the defect of *elo-5*(*lf*).

GC analyses of deletion mutants of *dhs-28* and *daf-22* showed that eliminating the activity of either gene did not cause significant increase in C17ISO level, suggesting that mutating these two genes has a weaker effect on the β-oxidation pathway than the *maoc-1* mutant. One possible scenario is that there is genetic redundancy associated with all of these genes so that none of these individual mutations completely abolish peroxisomal β-oxidation function and thus cannot sufficiently raise the mmBCFA level for the suppression of *elo-5*(*lf*). Consistent with this idea, none of three mutants displayed robust morphological defects (see further discussion in Discussion). An RNAi-based search for suppression of the *elo-5*(*lf*) phenotype failed to identify factors in additional pathways potentially involved in FA degradation, although it is possible that RNAi may not be sufficient to disrupt the function of some peroxisomal genes.

Together, these results suggest that the increase of mmBCFA level in the *prx-5*(*lf*)*; elo-5*(*lf*) suppressor mutant is not likely caused by a defect in a single peroxisome-involved mechanism that impacts mmBCFA degradation or biosynthesis. While β-oxidation may contribute to the change in mmBCFA level, β-oxidation-independent mechanisms are also likely to be involved (see Discussion).

## Discussion

Despite the broad presence of mmBCFAs in multiple organisms, little is known about their metabolism and roles in regulating development. In our previous studies, we discovered that inactivating the *elo-5* gene depleted mmBCFAs in *C. elegans*, causing robust arrest in post-embryonic development [11,14]. A mmBCFA-containing sphingolipid mediates this function by activating the TORC1 signaling pathway in the intestine [16,33]. In this study, we identified a new physiological role for peroxisomal functions in regulating mmBCFA metabolism that critically affects animal development and behaviors. Compromising the peroxisomal import function leads to an increase in mmBCFA levels in animals defective in mmBCFA biosynthesis, and likely does so by affecting multiple mechanisms. While over-expression of *elo-6* is not sufficient to suppress *elo-5*(*lf*), we cannot exclude the possibility that *prx-5*(*lf*) increases the synthesis of mmBCFA in the *elo-5*(*lf*) mutant even though it is unlikely given the known roles associated with the peroxisome.

Fatty acid β-oxidation is one of the important peroxisomal functions in all eukaryotic organisms. Compromising peroxisomal FA β-oxidation function has been shown to cause acyl-CoA accumulation [34]. The results from this study suggest that *prx-5* mutants have a reduced rate of mmBCFA degradation. In the *elo-5*(*lf*) mutant, although mmBCFA biosynthesis is dramatically compromised, it still remains at a low level, which depends on the function of *elo-6*. Under such a condition, decreasing mmBCFA degradation through compromising peroxisomal FA β-oxidation might increase the impermissibly low concentration of mmBCFAs and thus rescue *elo-5*(*lf*) developmental defect phenotype. However, we failed to phenocopy the suppression effect of *elo-5*(*lf*) by mutating or knocking down genes responsible for peroxisomal fatty acid β-oxidation. The possible explanation is that an inactivating *prx-5* compromises multiple pathways involved in fatty acid degradation, including the peroxisomal FA β-oxidation pathway and other lipid synthesis pathway. Compromising peroxisomal FA β-oxidation alone may contribute to, but may not be sufficient, to cause the adequate increase in mmBCFA level that suppresses the developmental arrest of *elo-5*(*lf*) observed in the *prx-5*(*lf*)*; elo-5*(*lf*) double mutant. Consistent with this idea, the level of mmBCFA C17ISO has mild increase in a deletion mutant of a key gene in the peroxisomal FA β-oxidation pathway (Figure 4C). It is possible that the RNAi and mutations of the each of three genes in the peroxisomal FA β-oxidation pathway only partially reduced the activity of FA oxidation due to redundant functions from other genes. However, because there are no obvious paralogs of these genes, the genetic redundancy, if it exists, would likely be due to functions of structurally unrelated genes with different biochemical roles, which makes it difficult to evaluate such a hypothesis.

It has been shown that, compared to mitochondrial FA β-oxidation that totally degrades FAs to generate energy, peroxisomal FA β-oxidation shortens the length of FA with limited cycles of β-oxidation and lower efficiency [31]. Additionally, peroxisomal FA β-oxidation has been shown to be involved in the synthesis of certain lipids such as components of active dauer pheromone [34,35]. We could thus also speculate that mmBCFAs might be partially degraded or chain-shortened in the peroxisome and integrated into certain types of complex lipids that negatively regulate post-embryonic development. An alternative hypothesis is that mmBCFA could be incorporated into other lipids by peroxisomal enzymes during lipid anabolism. This would reduce mmBCFA levels and compete with their developmental roles. Therefore, compromising peroxisomal function may decrease the efflux of mmBCFA and lead to increasing the level of mmBCFAs.

Thus, our results raised several interesting and important questions regarding peroxisome-mmBCFA relations and functions. For example, what other peroxisomal pathways are involved in mmBCFA metabolism, and are such mechanisms involved in feedback regulation of mmBCFA levels. It is certainly important for us to understand whether the findings in *C. elegans* are conserved in mammals and if so what are physiological effects of *de novo* biosynthesis and dietary uptake of mmBCFA in humans.

## References

[1] Spector AA, Yorek MA (1985) Membrane lipid composition and cellular function. J Lipid Res 26: 1015-1035. PubMed: 3906008. 3906008

[2] Yeagle PL (1989) Lipid regulation of cell membrane structure and function. FASEB J 3: 1833-1842. PubMed: 2469614. 2469614

[3] Wymann MP, Schneiter R (2008) Lipid signalling in disease. Nat Rev Mol Cell Biol 9: 162-176. doi:10.1038/nrg2324. PubMed: 18216772. 18216772 10.1038/nrm2335

[4] Pitson SM (2011) Regulation of sphingosine kinase and sphingolipid signaling. Trends Biochem Sci 36: 97-107. doi:10.1016/j.tibs.2010.08.001. PubMed: 20870412. 20870412 10.1016/j.tibs.2010.08.001

[5] Jordan SD, Könner AC, Brüning JC (2010) Sensing the fuels: glucose and lipid signaling in the CNS controlling energy homeostasis. Cell Mol Life Sci 67: 3255-3273. doi:10.1007/s00018-010-0414-7. PubMed: 20549539. 20549539 10.1007/s00018-010-0414-7 PMC2933848

[6] Vrablik TL, Watts JL (2012) Emerging roles for specific fatty acids in developmental processes. Genes Dev 26: 631-637. doi:10.1101/gad.190777.112. PubMed: 22474257. 22474257 10.1101/gad.190777.112 PMC3323873

[7] Webster CM, Deline ML, Watts JL (2013) Stress response pathways protect germ cells from omega-6 polyunsaturated fatty acid-mediated toxicity in Caenorhabditis elegans. Dev Biol 373: 14-25. doi:10.1016/j.ydbio.2012.10.002. PubMed: 23064027. 23064027 10.1016/j.ydbio.2012.10.002 PMC3508147

[8] Vrablik TL, Watts JL (2013) Polyunsaturated fatty acid derived signaling in reproduction and development: insights from Caenorhabditis elegans and Drosophila melanogaster. Mol Reprod Dev 80: 244-259. doi:10.1002/mrd.22167. PubMed: 23440886. 23440886 10.1002/mrd.22167 PMC4350910

[9] Heird WC, Lapillonne A (2005) The role of essential fatty acids in development. Annu Rev Nutr 25: 549-571. doi:10.1146/annurev.nutr.24.012003.132254. PubMed: 16011478. 16011478 10.1146/annurev.nutr.24.012003.132254

[10] Riediger ND, Othman RA, Suh M, Moghadasian MH (2009) A systemic review of the roles of n-3 fatty acids in health and disease. J Am Diet Assoc 109: 668-679. doi:10.1016/j.jada.2008.12.022. PubMed: 19328262. 19328262 10.1016/j.jada.2008.12.022

[11] Kniazeva M, Crawford QT, Seiber M, Wang CY, Han M (2004) Monomethyl branched-chain fatty acids play an essential role in Caenorhabditis elegans development. PLOS Biol 2: E257. doi:10.1371/journal.pbio.0020257. PubMed: 15340492. 15340492 10.1371/journal.pbio.0020257 PMC514883

[12] Jones LN, Rivett DE (1997) The role of 18-methyleicosanoic acid in the structure and formation of mammalian hair fibres. Micron 28: 469-485. doi:10.1016/S0968-4328(97)00039-5. PubMed: 9519472. 9519472 10.1016/s0968-4328(97)00039-5

[13] Hradec J, Dufek P (1994) Determination of cholesteryl 14-methylhexadecanoate in blood serum by reversed-phase high-performance liquid chromatography. J Chromatogr B Appl Biomed 660: 386-389. PubMed: 7866530. 10.1016/0378-4347(94)00292-4 7866530

[14] Kniazeva M, Euler T, Han M (2008) A branched-chain fatty acid is involved in post-embryonic growth control in parallel to the insulin receptor pathway and its biosynthesis is feedback-regulated in C. elegans. Genes Dev 22: 2102-2110. doi:10.1101/gad.1692008. PubMed: 18676815. 18676815 10.1101/gad.1692008 PMC2492746

[15] Kniazeva M, Shen H, Euler T, Wang C, Han M (2012) Regulation of maternal phospholipid composition and IP(3)-dependent embryonic membrane dynamics by a specific fatty acid metabolic event in C. elegans. Genes Dev 26: 554-566. doi:10.1101/gad.187054.112. PubMed: 22426533. 22426533 10.1101/gad.187054.112 PMC3315117

[16] Zhu H, Shen H, Sewell AK, Kniazeva M, Han M (2013) A novel sphingolipid-TORC1 pathway critically promotes postembryonic development in Caenorhabditis elegans. eLife. 2: e00429. 23705068 10.7554/eLife.00429 PMC3660743

[17] Thieringer H, Moellers B, Dodt G, Kunau WH, Driscoll M (2003) Modeling human peroxisome biogenesis disorders in the nematode Caenorhabditis elegans. J Cell Sci 116: 1797-1804. doi:10.1242/jcs.00380. PubMed: 12665560. 12665560 10.1242/jcs.00380

[18] Purdue PE, Lazarow PB (2001) Peroxisome biogenesis. Annu Rev Cell Dev Biol 17: 701-752. doi:10.1146/annurev.cellbio.17.1.701. PubMed: 11687502. 11687502 10.1146/annurev.cellbio.17.1.701

[19] Moser HW (1999) Genotype-phenotype correlations in disorders of peroxisome biogenesis. Mol Genet Metab 68: 316-327. doi:10.1006/mgme.1999.2926. PubMed: 10527683. 10527683 10.1006/mgme.1999.2926

[20] Seamen E, Blanchette JM, Han M (2009) P-type ATPase TAT-2 negatively regulates monomethyl branched-chain fatty acid mediated function in post-embryonic growth and development in C. elegans. PLOS Genet 5: e1000589. 19662161 10.1371/journal.pgen.1000589 PMC2716530

[21] Davis MW, Hammarlund M, Harrach T, Hullett P, Olsen S et al. (2005) Rapid single nucleotide polymorphism mapping in C. elegans. BMC Genomics 6: 118. doi:10.1186/1471-2164-6-118. PubMed: 16156901. 16156901 10.1186/1471-2164-6-118 PMC1242227

[22] Hillier LW, Marth GT, Quinlan AR, Dooling D, Fewell G et al. (2008) Whole-genome sequencing and variant discovery in C. elegans. Nat Methods 5: 183-188. doi:10.1038/nmeth.1179. PubMed: 18204455. 18204455 10.1038/nmeth.1179

[23] Bigelow H, Doitsidou M, Sarin S, Hobert O (2009) MAQGene: software to facilitate C. elegans mutant genome sequence analysis. Nat Methods 6: 549. doi:10.1038/nmeth.f.260. PubMed: 19620971. 19620971 10.1038/nmeth.f.260 PMC2854518

[24] Kamath RS, Fraser AG, Dong Y, Poulin G, Durbin R et al. (2003) Systematic functional analysis of the Caenorhabditis elegans genome using RNAi. Nature 421: 231-237. doi:10.1038/nature01278. PubMed: 12529635. 12529635 10.1038/nature01278

[25] Zipperlen P, Fraser AG, Kamath RS, Martinez-Campos M, Ahringer J (2001) Roles for 147 embryonic lethal genes on C. elegans chromosome I identified by RNA interference and video microscopy. EMBO J 20: 3984-3992. doi:10.1093/emboj/20.15.3984. PubMed: 11483502. 11483502 10.1093/emboj/20.15.3984 PMC149177

[26] Rebbapragada I, Lykke-Andersen J (2009) Execution of nonsense-mediated mRNA decay: what defines a substrate? Curr Opin Cell Biol 21: 394-402. doi:10.1016/j.ceb.2009.02.007. PubMed: 19359157. 19359157 10.1016/j.ceb.2009.02.007

[27] Steinberg SJ, Dodt G, Raymond GV, Braverman NE, Moser AB et al. (2006) Peroxisome biogenesis disorders. Biochim Biophys Acta 1763: 1733-1748. doi:10.1016/j.bbamcr.2006.09.010. PubMed: 17055079. 17055079 10.1016/j.bbamcr.2006.09.010

[28] Brock TJ, Browse J, Watts JL (2006) Genetic regulation of unsaturated fatty acid composition in C. elegans. PLOS Genet 2: e108. doi:10.1371/journal.pgen.0020108. PubMed: 16839188. 16839188 10.1371/journal.pgen.0020108 PMC1500810

[29] Watts JL, Browse J (2002) Genetic dissection of polyunsaturated fatty acid synthesis in Caenorhabditis elegans. Proc Natl Acad Sci U S A 99: 5854-5859. doi:10.1073/pnas.092064799. PubMed: 11972048. 11972048 10.1073/pnas.092064799 PMC122866

[30] Zhang Y, Zou X, Ding Y, Wang H, Wu X et al. (2013) Comparative genomics and functional study of lipid metabolic genes in Caenorhabditis elegans. BMC Genomics 14: 164. doi:10.1186/1471-2164-14-164. PubMed: 23496871. 23496871 10.1186/1471-2164-14-164 PMC3602672

[31] Poirier Y, Antonenkov VD, Glumoff T, Hiltunen JK (2006) Peroxisomal beta-oxidation--a metabolic pathway with multiple functions. Biochim Biophys Acta 1763: 1413-1426. doi:10.1016/j.bbamcr.2006.08.034. PubMed: 17028011. 17028011 10.1016/j.bbamcr.2006.08.034

[32] Zhang SO, Box AC, Xu N, Le Men J, Yu J et al. (2010) Genetic and dietary regulation of lipid droplet expansion in Caenorhabditis elegans. Proc Natl Acad Sci U S A 107: 4640-4645. doi:10.1073/pnas.0912308107. PubMed: 20176933. 20176933 10.1073/pnas.0912308107 PMC2842062

[33] Zhu H, Shen H, Sewell AK, Kniazeva M, Han M (2013) A novel sphingolipid-TORC1 pathway critically promotes postembryonic development in C. elegans. eLife. In press 10.7554/eLife.00429 PMC3660743 23705068

[34] Joo HJ, Yim YH, Jeong PY, Jin YX, Lee JE et al. (2009) Caenorhabditis elegans utilizes dauer pheromone biosynthesis to dispose of toxic peroxisomal fatty acids for cellular homoeostasis. Biochem J 422: 61-71. doi:10.1042/BJ20090513. PubMed: 19496754. 19496754 10.1042/BJ20090513

[35] Butcher RA, Ragains JR, Li W, Ruvkun G, Clardy J et al. (2009) Biosynthesis of the Caenorhabditis elegans dauer pheromone. Proc Natl Acad Sci U S A 106: 1875-1879. doi:10.1073/pnas.0810338106. PubMed: 19174521. 19174521 10.1073/pnas.0810338106 PMC2631283

